# P44 A service evaluation of clinician interpretation of microbiology reports at two different hospital trusts in the form of an educational survey during World Antimicrobial Resistance Awareness Week

**DOI:** 10.1093/jacamr/dlag102.050

**Published:** 2026-06-26

**Authors:** Christine Agbenu, Sumita K Pai, James Honey-Shastan, Dennis A Mlangeni, Netta Tyler, Cristiano Serra, Ruth M Kappeler

**Affiliations:** North West Anglia NHS Foundation Trust, Huntingdon, UK; Royal Papworth Hospital NHS Foundation Trust, Cambridge, UK; North West Anglia NHS Foundation Trust, Peterborough, UK; North West Anglia NHS Foundation Trust, Peterborough, UK; Royal Papworth Hospital NHS Foundation Trust, Cambridge, UK; Royal Papworth Hospital NHS Foundation Trust, Cambridge, UK; Royal Papworth Hospital NHS Foundation Trust, Cambridge, UK

## Abstract

**Background:**

Accurate interpretation of microbiology results is essential for ensuring appropriate antimicrobial therapy, optimizing patient outcomes, and minimizing the development of antimicrobial resistance. Misinterpretation can lead to inappropriate prescribing, including unnecessary use of broad-spectrum antibiotics, failure to de-escalate therapy, or treatment of colonization rather than true infection. Improving clinician understanding of microbiology reporting is therefore a key component of antimicrobial stewardship programmes.

**Objectives:**

This service evaluation aimed to assess clinicians’ interpretation of microbiology reports and their associated antimicrobial prescribing decisions across two hospital trusts. A secondary aim was to identify specific areas requiring improvement in reporting practices or clinician education, and to inform targeted interventions to optimize infection management.

**Methods:**

A survey was developed with involvement from microbiology consultants in two different trusts, specialist infection pharmacists and a resident doctor in foundation training. The survey collected some general demographic information, including grade/position, specialty, years of experience and trust. It then assessed knowledge and clinical decision-making using a series of single best answer and multiple-response questions based on common microbiology reporting scenarios. The survey was distributed via email lists, informal clinician communication channels (including WhatsApp), and an educational stall during World Antimicrobial Resistance Awareness Week. Data were analysed using descriptive statistics, with thematic evaluation of performance across key domains. Microbiology report formats at each trust were also reviewed to identify potential areas for improvement.

**Results:**

A total of 45 clinicians responded (17 from Trust A, a tertiary referral centre and 28 from Trust B, a district general hospital trust), representing a range of specialties and experience levels. Respondents included 18 pharmacists, while all five consultant respondents were from Trust A. Key themes identified included interpretation of antimicrobial susceptibilities, antimicrobial stewardship, recognition of when cultures require treatment, infection control principles, antibiotic-specific risks and calling microbiology. While most respondents correctly interpreted ‘susceptible’ and ‘resistant’ results, in one scenario only 29% selected appropriate higher dose of piperacillin–tazobactam when the susceptibility was reported as ‘Intermediate’, with 49% opting for broader-spectrum meropenem. Encouragingly, 65% avoided unnecessary IV meropenem in a separate scenario, favouring oral options or microbiology advice. Recognition of situations not requiring treatment was generally strong: 78% correctly identified urinary catheter-associated mixed growth as colonization, and 60% recognized a likely blood culture contaminant. Appropriate management of MRSA in wound cultures was identified by 67% of respondents. Knowledge of infection control measures was high, with over 80% correctly identifying the need for patient isolation and escalation to infection control teams. However, some prescribing challenges remained, including suboptimal antibiotic selection, with 38% choosing clindamycin where more appropriate alternatives existed.

**Conclusions:**

Despite limitations including small sample size and potential selection bias—partly due to timing during industrial action—this evaluation identified both strengths and areas for improvement in clinician interpretation of microbiology reports. While awareness of key antimicrobial stewardship principles is evident, further education is required to support more nuanced clinical decision-making and reduce unnecessary broad-spectrum antibiotic use. Future interventions may include targeted educational sessions and enhanced microbiology report design—particularly clearer interpretation of intermediate (‘I’) susceptibilities.

